# Soluble klotho regulates the function of salivary glands by activating KLF4 pathways

**DOI:** 10.18632/aging.102318

**Published:** 2019-10-02

**Authors:** Nguyen Chi Tai, Soo-A Kim, Sang-Gun Ahn

**Affiliations:** 1 Department of Pathology, School of Dentistry, Chosun University, Gwangju 61452, Republic of Korea; 2 Department of Biochemistry, School of Oriental Medicine, Dongguk University, Gyeongju 38066, Republic of Korea

**Keywords:** soluble klotho, salivary gland, KLF4, aging

## Abstract

The dysfunction of salivary glands commonly induces dry mouth, infections, and dental caries caused by a lack of saliva. This study was performed to determine the genetic and functional changes in salivary glands using a klotho (-/-) mouse model. Here, we confirmed the attenuation of KLF4 expression in the salivary glands of klotho (-/-) mice. Soluble klotho overexpression induced KLF4 transcription and KLF4-mediated signaling pathways, including mTOR, AMPK, and SOD1/2. Silencing klotho via siRNA significantly down-regulated KLF4 expression. Additionally, we monitored the function of salivary glands and soluble klotho and/or KLF4 responses and demonstrated that soluble klotho increased the expression of KLF4 and markers of salivary gland function (α-amylase, ZO-1, and Aqua5) in primary cultured salivary gland cells from wild type and klotho (-/-) mice. In a 3D culture system, cell sphere aggregates were observed in soluble klotho- or KLF4-expressing cells and exhibited higher expression levels of salivary gland function-related proteins than those in nontransfected cells. These results suggest that activation of the klotho-mediated KLF4 signaling pathway contributes to potentiating the function of salivary glands.

## INTRODUCTION

Salivary gland dysfunctional changes occur with reduced salivary flow and dry mouth (xerostomia) and commonly involve oral dysfunction, tooth structure deterioration, and infection through reduced salivation [1, 2]. Anatomically, aging induces atrophy of acinar cells (ACs) and replacement of normal gland parenchyma with adipose tissue, connective tissue, and oncocytes [3, 4]. Recently, numerous medical drugs and treatments (radiation, chemotherapy) have been shown to significantly contribute to salivary gland dysfunction [5, 6]. Although changes associated with salivary gland dysfunction are affected by multiple factors, such as the environment, not all changes are considered to be physiologic, and how aging influences the function of salivary glands is unclear.

Klotho is a transmembrane protein and a putative antiaging agent. Klotho-knockout mice demonstrate age-related phenotypes, such as a short lifespan, growth retardation, infertility, skin atrophy, hypoglycemia, hyposalivation, hyperphosphatemia, ectopic calcification, osteoporosis and pulmonary emphysema [7, 8].

The membrane klotho type acts as a coreceptor of fibroblast growth factor 23 (FGF23) and is capable of regulating phosphate and vitamin D metabolism [9, 10]. FGF23 and klotho signaling prevent tissue atrophy by stimulating cell proliferation and preventing vitamin D-induced cell death by apoptosis. The soluble klotho type circulates throughout the body via blood, urine and cerebrospinal fluid and expresses hormone-like functions, including signaling factor repression, oxidative stress suppression and transporter regulation. The anti-aging properties of klotho protein are also attributed to the cytoprotective effect of soluble klotho [11]. Soluble klotho inhibits the tyrosine phosphorylation of insulin and the IGF-1 receptor [12]. By inhibiting insulin/IGF-1 signaling pathway, soluble klotho also inhibits Fork head box O1 (FOXO1) phosphorylation and allows FOXO1 to bind mitochondrial manganese-superoxide dismutase (SOD2), an antioxidant enzyme, thus increasing the cellular resistance to oxidative stress by reactive oxygen species [13]. In human vascular endothelial cells, soluble klotho inhibits the caspase-3/caspase-9 and p53/p21 pathways, thus reducing the H_2_O_2_-induced apoptosis and senescence [14]. Additionally, soluble klotho binds to Wnt ligands and the type II receptor of TGF and inhibits their ability to induce Wnt/β-catenin and/or TGF-β1 signaling [15, 16].

In our previous study, we analyzed the gene expression profiles of salivary glands from klotho-deficient mice (klotho -/-). Microarray analysis showed that the expression levels of alpha2-Na^+^/K^+^-ATPase (Atp1a2), Ca^2+^-ATPase (Atp2a1), epidermal growth factor (EGF), and nerve growth factor (NGF), which have been suggested to regulate submandibular salivary gland function, were significantly decreased. In a network constructed by the differentially expressed genes induced by klotho depletion, proliferator-activated receptor-γ (PPARγ), which regulates energy homeostasis and insulin sensitivity, was located at the core [17]. Recently, we found that soluble klotho induces the expression of Kruppel-like factor 4 (KLF4) using a transcription factor assay (unpublished data). However, how soluble klotho exerts these protective effects on aged salivary gland dysfunction is poorly understood.

Therefore, this study aimed to evaluate salivary gland functional changes by the soluble klotho/KLF4 signaling pathway, and to provide further baseline information for preclinical research on salivary gland dysfunction related to aging.

## RESULTS

### KLF4 expression in klotho (-/-) mouse organs

**Figure 1:**
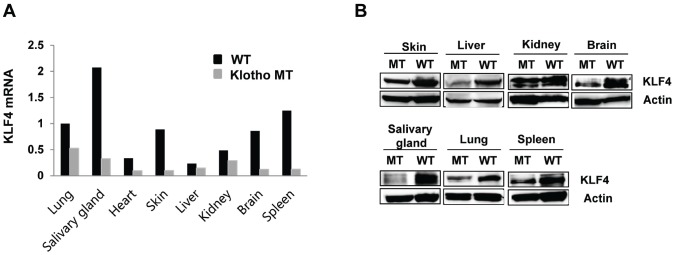
**Kruppel-like factor (KLF) 4 is downregulated in various tissues harvested from klotho (-/-) mice.** (**A**) Expression of mRNA encoding KLF4 in wild type and klotho (-/-) mouse tissues (as measured by qRT-PCR). (**B**) Protein expression of KLF4 in tissues harvested from wild-type and klotho (-/-) mice. Total protein samples were collected from the skin, liver, kidney, brain, salivary gland, lung, and spleen. The expression of KLF4 protein was determined by Western blot. Actin was used as an internal control.

To determine the tissue level expression of KLF in klotho (-/-) mice, we analyzed KLF4 expression in 8 organs (lung, salivary gland, heart, skin, liver, kidney, brain, spleen) harvested from four-week-old klotho (-/-) mice. Quantitative RT-PCR revealed that the mRNA levels of KLF4 were decreased to different extents—from 2- to 7-fold—in several organs. The salivary glands, skin, brain, and spleen exhibited significantly decreased KLF4 expression in klotho (-/-) mice compared to that in these organs of wild-type mice (Figure 1A). Similarly, KLF4 protein expression was also decreased in all tissues of klotho (-/-) mice (Figure 1B).

### Klotho induces KLF4 expression

**Figure 2:**
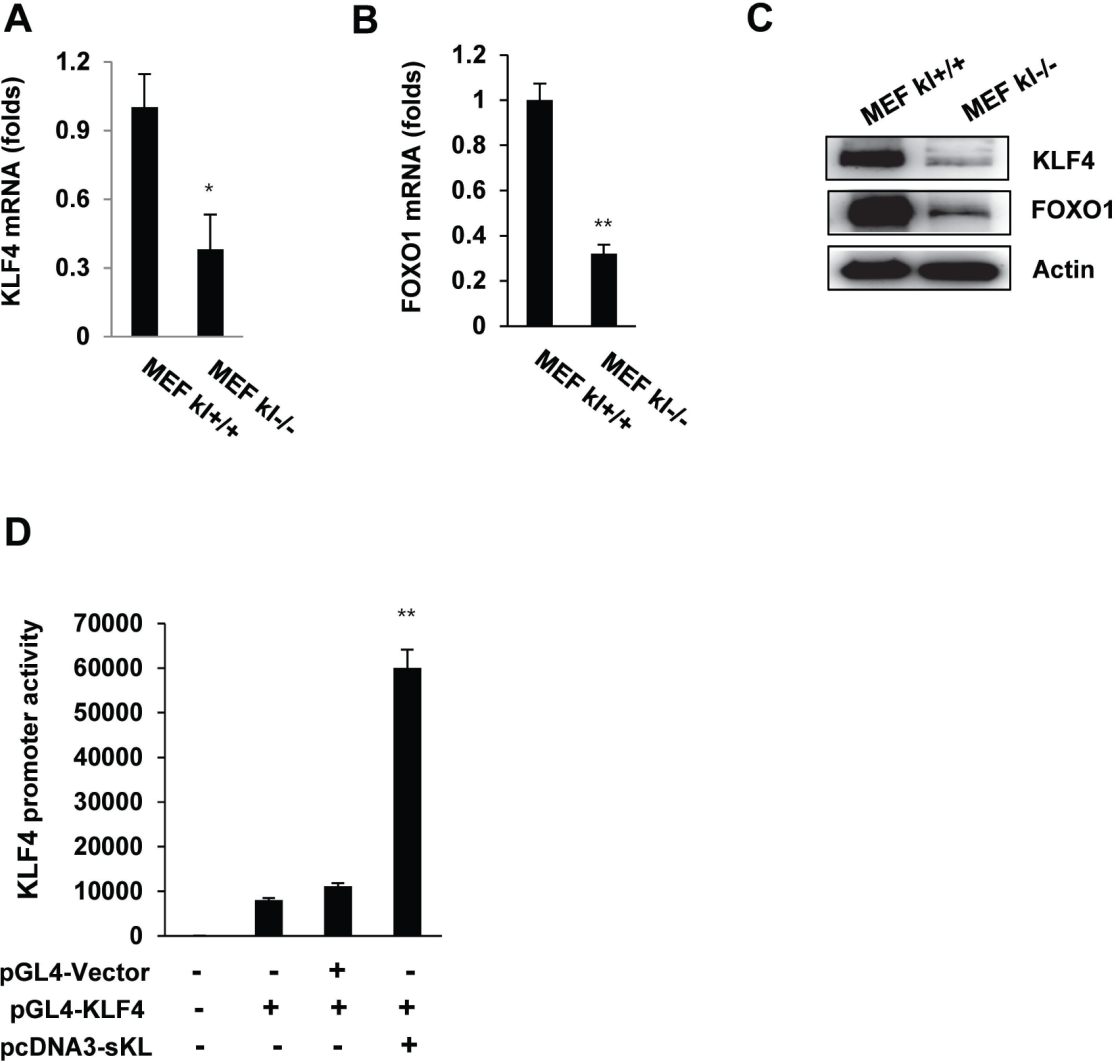
**The expression of KLF4 in wild-type and klotho (-/-) MEFs. qRT-PCR analysis of KLF4 and FOXO1 in wild-type and klotho (-/-) MEFs.** Total RNA samples were prepared from wild-type and klotho (-/-) MEFs, followed by quantitative RT-PCR analysis to examine the expression levels of KLF4 (**A**) and FOXO1 (**B**). Transcript abundances were normalized to the GAPDH RNA level and are expressed as relative values. The mean ± S.D. of three independent experiments is shown. (**C**) Western blot analysis was performed to assess the KLF4 and FOXO1 protein levels. (**D**) Soluble klotho induced KLF4 transcriptional activation. Transient cotransfection with the pGL3-KLF4 reporter construct and pcDNA3-soluble klotho was performed in HEK239 cells, followed by the dual-luciferase assay as described in the Materials and Methods. The mean ± S.D. of three independent experiments is shown (*p < 0.05, **p < 0.01).

To elucidate the relationship between klotho and KLF4 gene expression, we evaluated KLF4 mRNA expression using real-time quantitative RT-PCR in wild-type and klotho (-/-) mouse embryonic fibroblasts (MEFs). Depletion of klotho led to significantly diminished mRNA and protein levels of KLF4 (Figure 2A–2C), and the promoter activity of KLF4 was significantly increased after soluble klotho transfection. These results indicated that klotho leads to the induction of KLF4 transcription (Figure 2D).

### Soluble klotho induces the KLF4-related pathway

**Figure 3:**
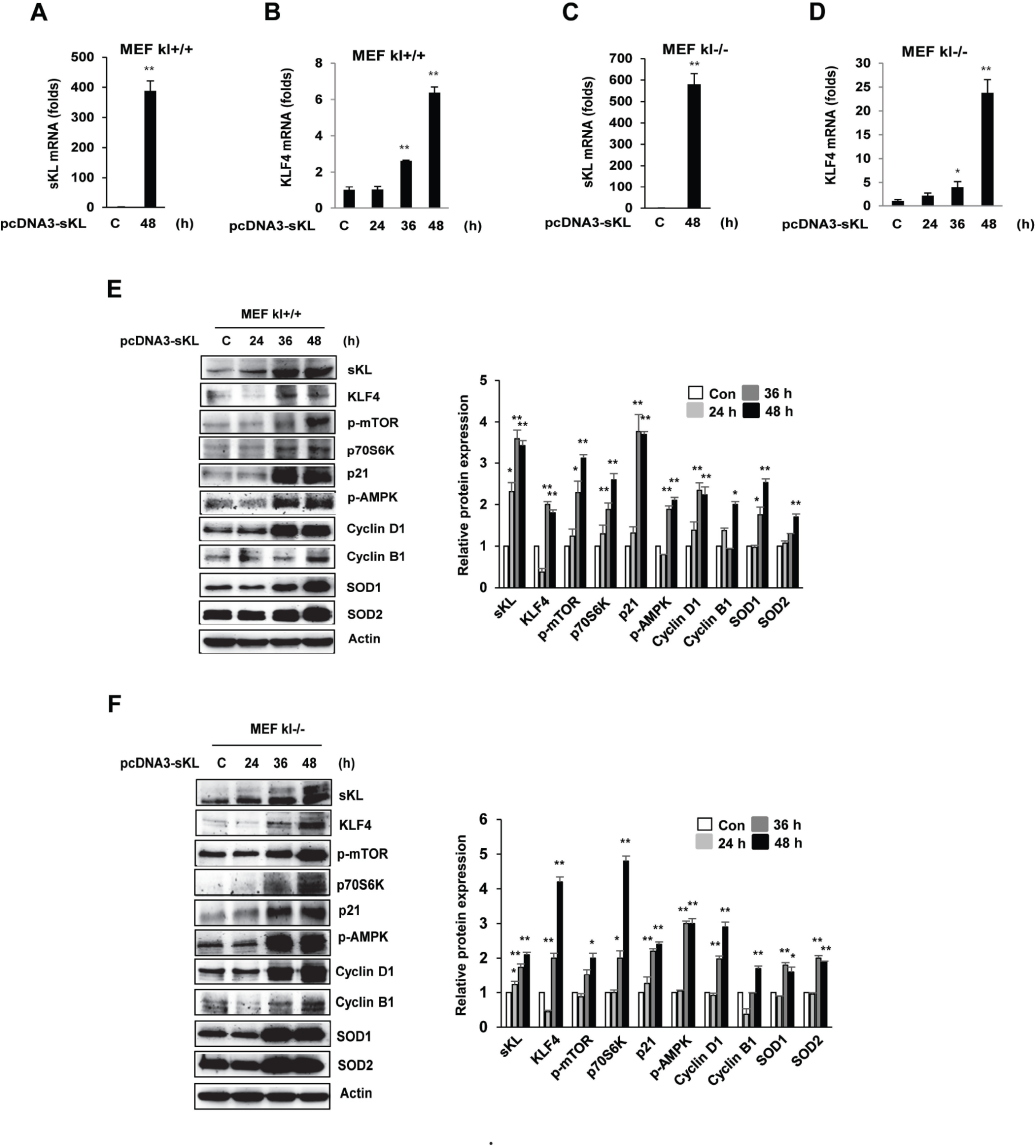
**Effects of soluble klotho on the expression of proteins belonging to the KLF4 pathway in wild-type and klotho (-/-) MEFs.** (**A**–**D**) qRT-PCR analysis of soluble klotho and KLF4. Total RNA samples were prepared from soluble klotho-transfected MEFs, and quantitative RT-PCR analysis was performed using the primers described. (**E**, **F**) The expression of proteins related to the KLF4 pathway. Wild-type and klotho (-/-) MEFs were transfected with soluble klotho expression plasmids (pcDNA3-soluble klotho). At 48 h after transfection, Western blot analysis was performed to assess the KLF4, mTOR, p70S6K, p21, AMPK, cyclin D1, cyclin B1, SOD1, and SOD2 levels. The mean ± S.D. of three independent experiments is shown (*p < 0.05, **p < 0.01).

To directly assess the functional contribution of soluble klotho to KLF4 signaling, we investigated the expression changes in KLF4-related genes upon the overexpression of soluble klotho in MEFs. We first evaluated the overexpression of soluble klotho in soluble klotho-transfected MEFs by real-time quantitative RT-PCR and observed abundant KLF4 mRNA expression (Figure 3A–3D). As shown in Figure 3E and 3F, soluble klotho the increased KLF4 protein expression in wild-type and klotho (-/-) MEFs. Immunoblotting analysis also revealed increased expression of KLF4-related genes, such as mTOR/p70s6k, p21, cyclinD1/cyclinB1, and SOD1/SOD2, in soluble klotho-transfected MEFs. The phosphorylation and/or expression of mTOR/p70s6k, p21, AMPK, cyclinD1, cyclinB1, SOD1, and SOD2 were strikingly upregulated in soluble klotho-transfected MEFs. In addition, the effects of soluble klotho protein on the expression of KLF4-related proteins are shown in Supplementary Figure 1 The upregulation of the KLF4 pathway by soluble klotho was further confirmed in HEK293 cells (Supplementary Figure 2).

### Soluble klotho and KLF4 regulate the p53/p21 and SOD1/2 pathways

**Figure 4:**
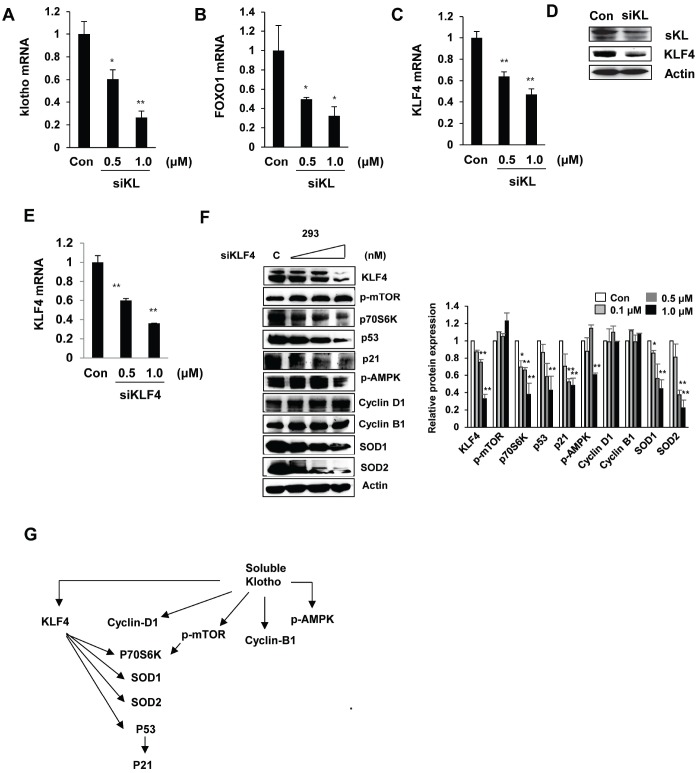
**Effects of si-klotho and siKLF4 on KLF4-related protein expression.** (**A**–**D**) Expression of klotho, FOXO-1, and KLF4 in si-klotho-overexpressing HEK293 cells. Cells were transfected with siRNA (0.5 or 1.0 nM) for 48 h. Comparisons of the si-klotho silencing efficiency by qRT-PCR and Western blot. (**E**) The KLF4 mRNA levels in HEK293 cells treated with KLF4 siRNA as measured by RT-PCR. (**F**) Western blot analysis of protein extracted from KLF4 siRNA (0.1, 0.5 or 1.0 nM)-transfected cells in a concentration-dependent manner. The expression levels of KLF4-related proteins, such as mTOR, p70S6K, p53, p21, AMPK, cyclin D1, cyclin B1, SOD1, SOD2, and actin (as a control), were determined. (**G**) Schematic diagram of the cell signaling pathway regulated by soluble klotho/KLF4. The mean ± S.D. of three independent experiments is shown (*p < 0.05, **p < 0.01).

We next assessed the effect of soluble klotho depletion on KLF4-related protein expression. The inhibition of soluble klotho by siRNA was detected by real-time PCR and Western blot analysis in HEK293 cells, and reduced expression of KLF4 and FOXO1 was observed. KLF4 protein expression was also inhibited in siRNA soluble klotho-transfected cells (Figure 4A–4D).

To further clarify whether KLF4 depletion modulates soluble klotho-induced KLF4 signaling, we knocked down KLF4 by siRNA in HEK293 cells. As shown in Figure 4E–4F, the expression of KLF4 was dramatically downregulated in siKLF4-transfected cells. Interestingly, the expression of SOD1, SOD2, and P53 was strikingly downregulated in siKLF4-transfected cells in a concentration-dependent manner. However, the soluble klotho-induced expression/activation of mammalian target of rapamycin (mTOR), cyclin D1, and cyclin B1 was not changed in siKLF4-transfected cells compared to that in control siRNA-transfected cells. Together, these results showed that soluble klotho directly regulates KLF4 expression and may modulate the cell cycle and antioxidant signaling by regulating p53/p21 and SOD1/2 through KLF4 signaling pathways (Figure 4G).

### Soluble klotho induces the function of primary salivary gland cells (PSGCs)

**Figure 5:**
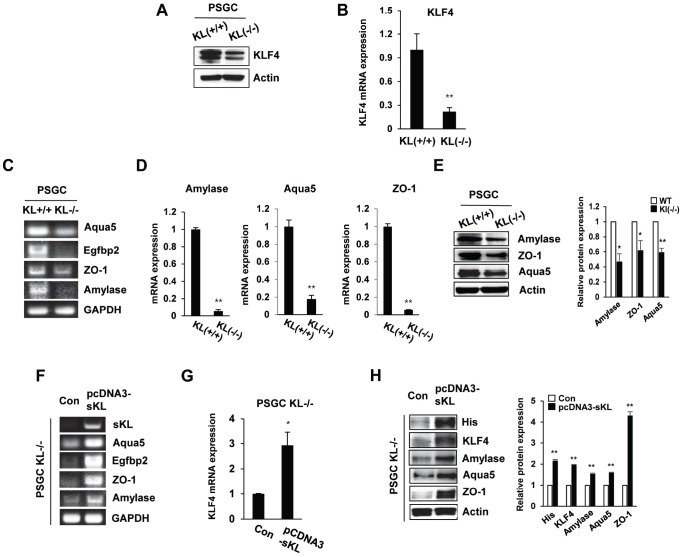
**The expression of salivary gland functional proteins in primary salivary gland cells (PSGCs).** (**A**, **B**) The protein and mRNA expression of KLF4 in KL(+/+) PSGCs and KL(-/-) PSGCs. (**C**–**E**) Comparison of functional protein or mRNA (aquaporin 5, EGFBP2, ZO-1, and amylase) expression between KL(+/+) PSGCs and KL(-/-) PSGCs by RT-PCR, qRT-PCR, and immunoblot analyses. (**F**–**H**) Effects of soluble klotho on functional protein expression. Cells were transfected with pcDNA3-soluble klotho for 48 h. Comparisons of salivary gland functional proteins by RT-PCR, qRT-PCR, and Western blot. The actin protein was used as an internal control. The results are reported as the mean ± SD (*p < 0.05, **p < 0.01).

Single-cell suspensions obtained by mechanical and enzymatic dissociation of klotho (-/-) mouse salivary glands were grown in our culture system as described in the Materials and Methods. In klotho (-/-) PSGCs, we examined the expression of KLF4 by real-time quantitative RT-PCR and Western blot. Similar to the results shown in Figure 1, KLF4 expression was decreased in klotho (-/-) PSGCs (Figure 5A and 5B).

To confirm the function of PSGC populations, we examined the expression of salivary gland functional markers, such as α-amylase (AC secretion product), aquaporin 5 (water channel protein), and ZO-1 (tight junction protein). Quantitative reverse transcription PCR (qRT-PCR) was used to validate the altered expression of these genes. Klotho (-/-) PSGCs showed reduced expression of α-amylase and aquaporin-5 compared to that in klotho (+/+) PSGCs. The tight junction protein ZO-1 was also inhibited in klotho (-/-) PSGCs (Figure 5C–5E). In contrast, the effect of soluble klotho overexpression on KLF4-related protein expression was further confirmed by RT-PCR. The expression of α-amylase, aquaporin-5, and ZO-1 was dramatically upregulated in soluble klotho-expressing klotho (-/-) PSGCs (Figure 5F–5H). Consistent with those in klotho (-/-) PSGCs, the mRNA levels of aquaporin 5 and EGFBP2 were increased in soluble klotho-transfected klotho (+/+) PSGCs. The mRNA expression of α-amylase, aquaporin-5, and ZO-1 in KLF4-overexpressing klotho (+/+) PSGCs was also confirmed by real-time quantitative RT-PCR. In addition, salivary gland functional marker expression in PSGCs was confirmed by Western blot analysis of soluble klotho- or KLF4-expressing wild-type PSGCs (Supplementary Figure 3).

### Soluble klotho-induced KLF4 induces differentiation into acinar-like cells

**Figure 6:**
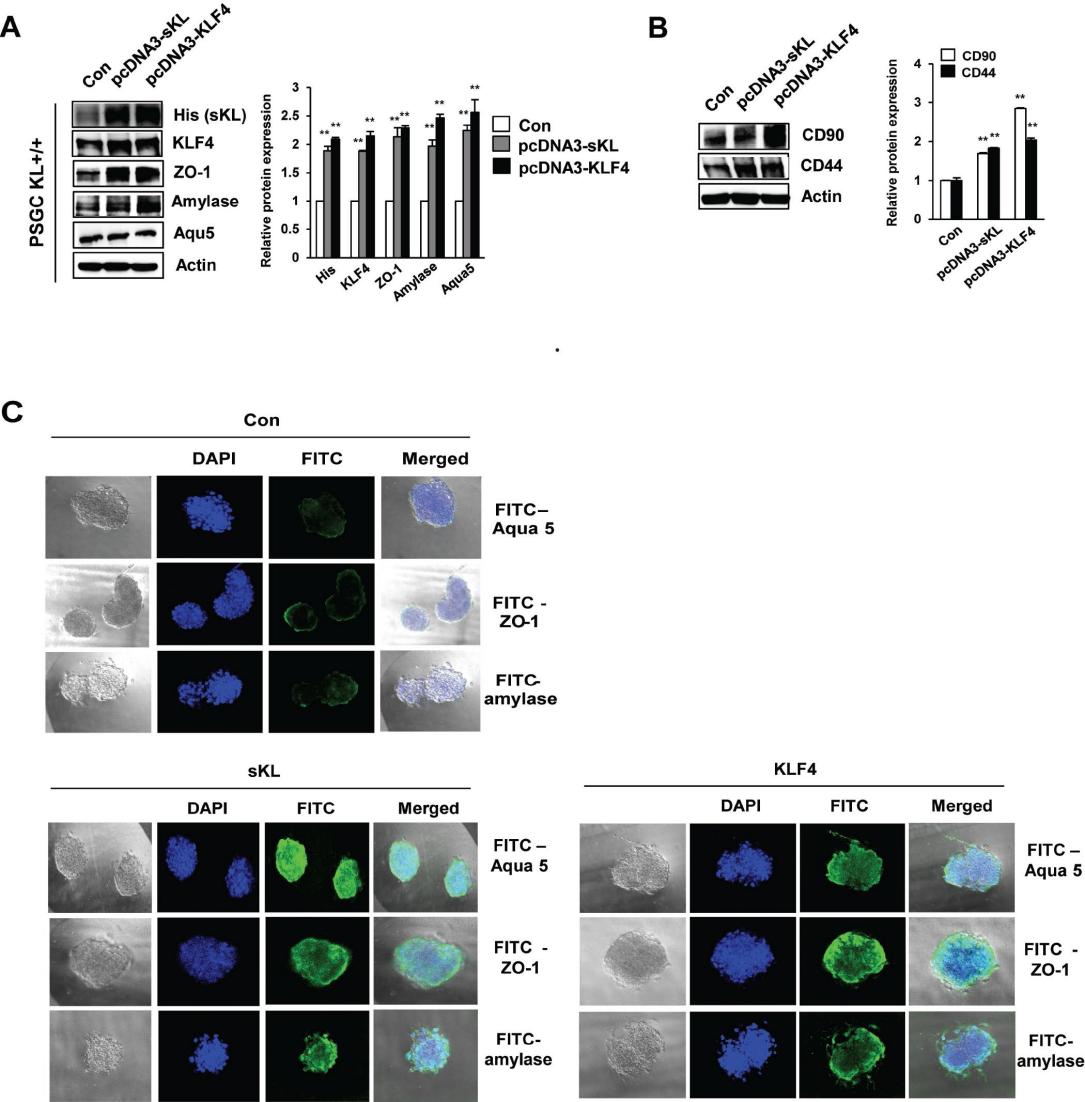
**Functional restoration of PSGCs by soluble klotho and KLF4 expression.** (**A**) Detection of the CD90 and CD44 protein levels in soluble klotho- or KLF4-expressing PSGCs. Cells were transfected with soluble klotho or KLF4 expression plasmids. At 48 h after transfection, total protein was prepared and subjected to immunoblotting. (**B**) Expression of KLF4, aquaporin 5, ZO-1, and amylase in PSGCs after transfection with soluble klotho or KLF4 expression plasmids. (**C**) Immunofluorescence staining of aquaporin 5, ZO-1, and amylase in PSGCs cultured on 3D Matrigel at day 5.

To determine whether PSGCs induced by soluble klotho or KLF4 transfection could differentiate into functional activating cells, such as salivary gland ACs, we utilized a 3D culture system. First, we observed the induction of typical mesenchymal stem cell (MSC) surface antigen markers, such as CD44 and CD90, which are known to interact with the extracellular matrix, in soluble klotho- or KLF4-expressing wild-type PSGCs (Figure 6A). In addition, salivary gland functional marker expression in soluble klotho- or KLF4-expressing wild-type PSGCs was confirmed by Western blot (Figure 6B). Then, 4D cultured PSGCs were immunostained for four AC markers, α-amylase, Aqua-5, and ZO-1, to confirm AC differentiation. Under soluble klotho or KLF4 transfection conditions, small colonies of epithelial-shaped cells were observed on the culture substrate within 5 days after seeding. Cells with a homogeneous polygonal shape proliferated and expanded to form an epithelial-like monolayer. The cells in the 3D culture substrate were generally covered by monolayered cells. In this crowded region, 3D cell aggregations and spherical shapes were observed (Figure 6C). As shown by immunofluorescence analysis of 3D cultured PSGCs, these spherical cells were positively labeled with AQP-5 and α-amylase. Notably, strong staining of ZO-1 was observed. These findings suggested that soluble klotho-induced PSGCs exhibited increased functional characteristics. The acceleration of the KLF4 pathway upon expression of soluble klotho or KLF4 was further confirmed in salivary gland ACs (Supplementary Figure 4).

## DISCUSSION

Soluble klotho has been suggested to function as a humoral factor that targets multiple tissues. A recent study demonstrated that soluble klotho attenuated the generation of reactive oxygen species by insulin and IGF-1 signaling [18, 19]. Soluble klotho also inhibited TGF-*β*-induced renal fibrosis and metastasis [20, 21] and blocked the levels of the inflammatory proteins TNF*α* and IFN*γ*. In addition, soluble klotho maintained a low plasma phosphate concentration by inhibiting both renal (NaPi-IIa) and intestinal (NaPi-IIb) phosphate transporters [22, 23]. Therefore, soluble klotho facilitates various cellular functions, including antiaging effects, and has tissue protective effects that are independent of FGF23/FGFR.

In a previous study, we performed a transcription factor (TF) activation plate array to profile the TF activities affected by soluble klotho expression. TFs play essential roles in regulating gene expression and act as sensors to monitor cellular changes and convert the signals into altered gene expression. We first found that KLF4 expression was significantly increased after soluble klotho transfection (unpublished data). In this study, KLF4 expression was clearly highly abundant and diverse in the salivary gland, followed by the lung, skin, brain and spleen. The high KLF4 expression in salivary glands is consistent with the immunoblot results. Based on this analysis, KLF4 expression was significantly downregulated in the salivary glands of klotho-deficient mice compared with that in wild-type mice. We confirmed that the expression of KLF4 was decreased in klotho (-/-) MEFs relative to that in wild-type cells. Moreover, using gene transfection and RNA interference techniques, we demonstrated that soluble klotho regulated the expression of KLF4. Interestingly, the KLF4 promoter activity was significantly increased in soluble klotho-overexpressing cells. These results strongly indicate that soluble klotho is involved in the regulation of KLF4 expression.

KLF4, an essential regulator of stem cells, was found to be specifically expressed in several normal tissues, especially the salivary gland. KLF4 is downregulated during parotid AC terminal differentiation [24]. We defined the expression profiles of KLF4-related proteins in soluble klotho-transfected cells, revealing that soluble klotho significantly increased the expression and/or activation of KLF4-regulated proteins, including mTOR, AMPK, p21, cyclin D1, cyclin B1, and SOD1/2. However, soluble klotho knockdown by siRNA significantly decreased the KLF4 mRNA and protein levels. In contrast, siRNA knockdown of KLF4 inhibited the cell cycle suppressors p53 and p21, the antioxidant enzymes AMPK and SOD1/2, and the mTOR target protein p70S6K but did not reduce mTOR phosphorylation or the cell cycle regulator proteins (cyclin D1 and cyclin B1) in the absence of KLF4, suggesting that mTOR activation and cyclin D1 and cyclin B1 expression depend on only klotho expression.

A previous study suggested that KLF4 primarily contributes to p53-dependent cell cycle arrest by DNA damage [25, 26]. KLF4 induces the cell cycle inhibitor p21^Cip1/Waf1^ by recruiting p53 [26]. KLF4 also inhibits the expression of the cell cycle regulators cyclin D1 and cyclin B1 [27, 28]. Thus, KLF4 is involved in cell cycle progression and maintains DNA integrity. However, siRNA - mediated knockdown of KLF4 did not affect the protein expression levels of cyclin D1 and cyclin B1. In contrast, we observed increased expression of cyclin D1 and cyclin B1 in soluble klotho-KLF4-induced cells. Our results suggest that although the expression of KLF4 is increased in soluble klotho-induced cells, KLF4 signaling is not directly involved in the expression of the cell cycle regulators cyclin D1 and cyclin B1 in these conditions. Based on these protein expression analysis results, we generated a regulatory pathway of soluble klotho with the profile of KLF4-regulated proteins. Our results suggest that KLF4 is induced by soluble klotho and strongly induces p53/p21, SOD1/2, and mTOR-independent p70S6K signaling.

We recently showed that submandibular salivary glands in klotho-deficient mice show a loss of granular ducts and mucous acini compared to those of wild-type mice [17]. Loss of granular ducts and mucous acini is believed to affect the function of ACs in salivary glands.

Therefore, we examined the critical role of soluble klotho/KLF4 in salivary gland dysfunction. In this study, we investigated the *in vitro* characteristics of PSGCs isolated from klotho wild-type and klotho (-/-) mice. The PSGCs of klotho (-/-) mice showed low KLF4 expression compared to those of wild-type mice and inhibited the expression of salivary gland functional markers, such as α-amylase, aquaporin 5, and ZO-1. However, overexpression of soluble klotho induced KLF4 and salivary gland functional markers in PSGCs. In addition, small colonies of epithelial-shaped cells were observed on the culture substrate within 5 days under soluble klotho or KLF4 transfection conditions. These spherical cells were positively labeled with AQP-5 and α-amylase. Notably, strong staining of ZO-1 was observed. The expression of salivary gland functional markers in soluble klotho- or KLF4-overexpressing ACs is shown in Supplementary Figure 4.

In addition, we demonstrated the expression of stem cell markers (CD44 and CD90) in soluble klotho-induced PSGCs. CD44, a stem cell marker, has previously been reported in serous ACs of human salivary gland tissue [29, 30]. Furthermore, CD44 was proposed to be involved in regulating the growth and renewal of normal salivary gland tissue [29]. Immunohistological analysis showed the expression of CD90 (Thy-1) in stromal cells in the periductal area of the salivary gland [31]. We hypothesize that primary salivary acinar-like cells that organize into acini-like spheroids *in vitro* will express CD44 and CD90 and retain these morphological and phenotypical characteristics after 3D culture. These results showed that soluble klotho-induced cells retain their ability to self-renew as stem cells and may restore the function of the salivary gland.

In summary, soluble klotho directly induces cyclin D1/B1 and AMPK and regulates p53/p21 and SOD1/2 signaling by inducing KLF4. Our findings suggest a critical role for soluble klotho/KLF4 in the functional restoration of the salivary gland and selectively inducing the proteins necessary for salivary gland function. Soluble klotho/KLF4 signaling in PSGCs induced the expression of salivary gland functional markers (α-amylase, aquaporin 5, and ZO-1). Additionally, 3D culture analysis showed that spherical cells were formed in soluble klotho- or KLF4-induced PSGCs. We predict that the expression of the progenitor cell markers CD44 and CD90 in soluble klotho-induced PSGCs may at least partially explain their regenerative potential. We expect that our results will contribute to better understanding soluble klotho signaling in the salivary gland and the development of potential cell therapies for patients with irreversible salivary gland function loss.

## MATERIALS AND METHODS

### Cell cultures

MEFs were derived and cultured from SAMP1/kl+/+ and SAMP1/kl-/- 13-day-old embryos that were obtained by mating SAMP1/kl+/- mice with SAMP1/kl+/- mice [32] and maintained in Dulbecco’s Modified Eagle’s Medium (DMEM, Gibco, NY) containing gentamycin, glutamine, MEM nonessential amino acids, 2-mercaptoethanol, and 10% fetal bovine serum (FBS, Gibco, NY). The human embryonic kidney 293 cell line and MEFs isolated from wild-type or klotho knockout mice were grown in DMEM supplemented with 10% FBS, 100 units/ml penicillin, and 100 μg/ml streptomycin (Invitrogen, CA) in a humidified 5% CO_2_/95% air atmosphere at 37°C. Immortalized human salivary gland ACs were cultured on keratinocyte serum-free medium (K-SFM, Gibco/Life Technologies, Grand Island, NY) containing 100 U/ml penicillin and 100 μg/ml streptomycin.

### Preparation of primary submandibular salivary gland cells from mice and *in vitro* culture

Submandibular gland tissues were harvested from 4- or 8-week-old wild-type and klotho (-/-) mice. The tissues were dissociated from cervical fascia and connective tissues under a dissecting microscope and gently collected in phosphate-buffered saline (PBS). Freshly dissociated tissues were washed twice with DMEM and Ham’s F-12 medium (DMEM/F12 medium, 1:1, Gibco, USA) supplemented with 100 U/mL penicillin G and 100 μg/ml streptomycin (Gibco), minced with scissors, and incubated with DMEM/F12 medium containing collagenase type II (0.025%), hyaluronidase (0.04%) and CaCl_2_ (6.25 mM) at 37°C for 40 min. Dissociated cells were centrifuged at 1000 rpm for 5 min, and the cell pellet was resuspended in the culture medium. The cells were plated in a 12-well plate at a density of 5 × 10^4^ cells/well in DMEM/F-12 (Gibco) medium supplemented with 100 U/mL penicillin G, 100 μg/ml streptomycin, 20 ng/mL EGF (Sigma-Aldrich), 20 ng/mL basic fibroblast growth factor (Gibco), 1/100 N2 supplement (Gibco), 10 μg of insulin-transferrin-selenium (Gibco), and 1 μM dexamethasone (Sigma-Aldrich) and incubated in 5% CO_2_ at 37°C. Fresh medium was added every three days. After 4-5 passages, the expression of salivary gland functional markers, such as α-amylase (AC secretion product), aquaporin 5 (water channel protein), and ZO-1 (tight junction protein), was examined.

### Real-time quantitative PCR

Total RNA was extracted with TRIzol reagent (Invitrogen). Reverse transcription was performed on 1 μg of total RNA using oligo dT primers and M-MLV Reverse Transcriptase (Invitrogen) in a final volume of 20 μl for 5 min at 65°C followed by 1 h at 37°C. Real-time quantitative PCR was performed using the SYBR Green kit (Thermo Fisher, USA) according to the manufacturer’s instructions. The expression levels of soluble klotho, KLF4, and FOXO1 mRNA were normalized to those of GAPDH for each sample. The following primers were used for real-time quantitative PCR detection: soluble klotho, 5′-GCTCTGCTTCCGCC ACTT-3′ (forward) and 5′-GCATGAGCCAGGAGGAG G-3′ (reverse); KLF4, 5′-TTCCAACTCGCTAACCCAC C-3′ (forward) and 5′-GCTCGGGACTCAGTGTAG-3′ (reverse); FOXO1, 5′-AGACCCGGGTTCTTTGACAC-3′ (forward) and 5′-TTGTCATAGGCTTCCCACCA-3′ (reverse); amylase, 5′-GGTGCAACAATGTTGGTGTC-3′ (forward) and 5′-ACTGCTTTGTCCAGCTTGAG-3′ (reverse); Aqua5, 5′-CGACCGTGTGGCTGTGGTCA-3′ (forward) and 5′-GTGCCGGTCAGTGTGCCGTC-3′ (reverse); ZO-1, 5′-CGAGGCATCATCCCAAATAAGA AC-3′ (forward) and 5′-TCCAGAAGTCTGCCCGATC AC-3′ (reverse). The samples were quantified based on the threshold cycle by interpolation from the standard curve. The reliability of the PCR results was evaluated by a solubility curve as follows: ΔCt = CT _(target gene)_ − CT _(internal reference)_ and ΔΔCt = ΔCt _(experimental group)_ − ΔCt _(control group)_. The relative expression levels of related genes were determined by the 2^−ΔΔCT^ method, and the experiments were repeated three times.

### siRNA interference

KLF4 siRNA was obtained in the form of Silencer Select Validated siRNA (Applied Biosystems). The sense sequence of the KLF4 siRNA was 5′-CUCGUUCUCG GCUUGUCUA (dTdT)-3′, and the antisense sequence was 5′-UAGACAAGCCGAGAACGAG (dTdT)-3′. The cells were transfected with siRNA (20 nM) using Lipofectamine 2000 siRNA Transfection Reagent (Invitrogen, Carlsbad, CA) according to the manufacturer’s instructions. The cells were harvested 48 h after transfection. Total cell lysates were separated by SDS-PAGE and analyzed by Western blot as described above.

### Western blot analysis

Cells were lysed, and proteins were extracted in a radio immunoprecipitation assay buffer (RIPA buffer, 25 mM Tris-HCl [pH 7.6], 150 mM NaCl, 1% NP-40, 1% sodium deoxycholate, 0.1% SDS; Pierce) and complete protease inhibitor cocktail (Sigma-Aldrich). The lysates were obtained after centrifugation, and the protein concentration was measured using the BCA protein assay kit (Pierce, Thermo Scientific). The lysates were separated by 12% SDS-PAGE. After transfer to PVDF membranes (Hybond-C; GE Amersham), the membranes were blocked for 1 h at room temperature with 5% nonfat dry milk in TBS-T buffer. The blocked membranes were then incubated with primary antibodies (1:1000 dilution) against klotho (10E2), KLF4 (3F3), FOXO1 (7G6C5), phospho-mTOR (D15C3), phospho-AMPK (D2E5), and p21 (D1F2), which were purchased from Cell Signaling Technology. The cyclin D1, cyclin B1, SOD1, SOD2, p70S6K, and actin antibodies were purchased from Santa Cruz Biotechnology (Santa Cruz, CA, USA). The membranes were washed 3 times with TBS-T buffer and then incubated with secondary antibodies conjugated to HRP (1:5,000 dilution) for 1 h at room temperature. The immunoreactive proteins were visualized with ECL detection reagents (Thermo).

### Luciferase activity assay

The pGL4-KLF4 promoter (-2.046 bp from the start codon) was generously provided by Dr. Yoshida (Jikei University, Tokyo, Japan). HEK293 cells were transfected with 1 μg of the pGL4-KLF4 Luc plasmid and 1 μg of Tk-Renilla by Fugene 6 HD (Promega) reagent and harvested after 48 h. Additionally, the cells were transfected with pcDNA3-sKL and/or pcDNA3-KLF4 using Fugene 6 HD reagent for 48 h. Transfected cells were lysed and measured with the Dual-Luciferase Reporter Assay System (Promega, WI, USA) according to the manufacturer’s instructions. Relative luciferase units were normalized by the Tk-Renilla luciferase value in each sample. Each experiment was performed in triplicate and repeated twice.

### Three-dimensional Matrigel spheroid cultures

One hundred microliters of Matrigel (Nitta Gelatin, Inc., Osaka, Japan; 8:1 Cellmatrix type-1: concentrated media) in 48-well chambers was allowed to solidify in a 37°C incubator for 1 h. Upon solidification, 5 x 10^4^ cells/well were seeded on Matrigel, and 3D spheroids were grown at 37°C in 5% CO_2_. For the generation of single-cell suspensions from 3D spheroids, spheres were isolated by removing media and adding 0.5 ml of Dispase (1 mg/ml) directly to the Matrigel followed by incubation for 1 h at 37°C in 5% CO_2_ to digest the Matrigel. The spheres were washed in PBS, centrifuged at 1200 rpm for 5 min, dissociated with TrypLE Express (Gibco) and passed through a 40 μm filter to remove clumps and obtain single-cell suspensions. The cells were then plated and grown as monolayers in either serum-free medium (SFM) or serum-containing medium (SCM).

### Immunofluorescence analysis

The presence of aquaporin-5 and ZO-1 in 3D cultured cells was confirmed by indirect immunofluorescence staining as described previously [33]. Briefly, samples were fixed with 10% formaldehyde/PBS for 10 min and permeabilized by PBS containing 1% BSA/0.2% Triton X-100 for 15 min at room temperature. The samples were incubated with primary antibodies against aquaporin-5 and ZO-1 (1:50 dilution) overnight at 4°C. After three washes with PBS/0.05% Tween 20, the samples were incubated with a secondary antibody (Alexa Fluor 488-conjugated goat anti-mouse IgG [H+L]; Invitrogen, CA), diluted 1:500 for 2 h at room temperature. The cell nuclei were counterstained by propidium iodide for 5 min and subsequently washed three times with PBS/0.05% Tween 20. The stained cells were visualized using a Nikon A1^+^ confocal microscope (Japan). The average intensity value of the nonspecifically bound secondary antibody was subtracted from all images using the ImageJ program.

### Statistical analysis

Data are presented as the mean ± SEM. Statistical significance between groups was determined by Student’s t test and one-way analysis of variance (ANOVA). *P*-values <0.05 were considered statistically significant.

## Supplementary Material

Supplementary Figures

## References

[1] Joaquin AM, Gollapudi S. Functional decline in aging and disease: a role for apoptosis. J Am Geriatr Soc. 2001; 49:1234–40. 10.1046/j.1532-5415.2001.04990.x 11559385

[2] Gupta A, Epstein JB, Sroussi H. Hyposalivation in elderly patients. J Can Dent Assoc. 2006; 72:841–46. 17109806

[3] Azevedo LR, Damante JH, Lara VS, Lauris JR. Age-related changes in human sublingual glands: a post mortem study. Arch Oral Biol. 2005; 50:565–74. 10.1016/j.archoralbio.2004.10.019 15848150

[4] Choi JS, Park IS, Kim SK, Lim JY, Kim YM. Analysis of age-related changes in the functional morphologies of salivary glands in mice. Arch Oral Biol. 2013; 58:1635–42. 10.1016/j.archoralbio.2013.07.008 24112729

[5] Wu AJ, Ship JA. A characterization of major salivary gland flow rates in the presence of medications and systemic diseases. Oral Surg Oral Med Oral Pathol. 1993; 76:301–06. 10.1016/0030-4220(93)90258-6 8378045

[6] Atkinson JC, Wu AJ. Salivary gland dysfunction: causes, symptoms, treatment. J Am Dent Assoc. 1994; 125:409–16. 10.14219/jada.archive.1994.0059 8176076

[7] Kuro-o M, Matsumura Y, Aizawa H, Kawaguchi H, Suga T, Utsugi T, Ohyama Y, Kurabayashi M, Kaname T, Kume E, Iwasaki H, Iida A, Shiraki-Iida T, et al. Mutation of the mouse klotho gene leads to a syndrome resembling ageing. Nature. 1997; 390:45–51. 10.1038/36285 9363890

[8] Matsumura Y, Aizawa H, Shiraki-Iida T, Nagai R, Kuro-o M, Nabeshima Y. Identification of the human klotho gene and its two transcripts encoding membrane and secreted klotho protein. Biochem Biophys Res Commun. 1998; 242:626–30. 10.1006/bbrc.1997.8019 9464267

[9] Kuro-O M. Klotho as a regulator of fibroblast growth factor signaling and phosphate/calcium metabolism. Curr Opin Nephrol Hypertens. 2006; 15:437–41. 10.1097/01.mnh.0000232885.81142.83 16775459

[10] Kuro-O M. Molecular mechanisms underlying accelerated aging by defects in the fgf23-klotho system. Int J Nephrol. 2018; 2018:9679841. 10.1155/2018/9679841 29951315 PMC5987335

[11] Kurosu H, Yamamoto M, Clark JD, Pastor JV, Nandi A, Gurnani P, McGuinness OP, Chikuda H, Yamaguchi M, Kawaguchi H, Shimomura I, Takayama Y, Herz J, et al. Suppression of aging in mice by the hormone Klotho. Science. 2005; 309:1829–33. 10.1126/science.1112766 16123266 PMC2536606

[12] Dalton GD, Xie J, An SW, Huang CL. New insights into the mechanism of action of soluble klotho. Front Endocrinol (Lausanne). 2017; 8:323. 10.3389/fendo.2017.00323 29250031 PMC5715364

[13] Kenyon C. The plasticity of aging: insights from long-lived mutants. Cell. 2005; 120:449–60. 10.1016/j.cell.2005.02.002 15734678

[14] Ikushima M, Rakugi H, Ishikawa K, Maekawa Y, Yamamoto K, Ohta J, Chihara Y, Kida I, Ogihara T. Anti-apoptotic and anti-senescence effects of Klotho on vascular endothelial cells. Biochem Biophys Res Commun. 2006; 339:827–32. 10.1016/j.bbrc.2005.11.094 16325773

[15] Mencke R, Olauson H, Hillebrands JL. Effects of Klotho on fibrosis and cancer: A renal focus on mechanisms and therapeutic strategies. Adv Drug Deliv Rev. 2017; 121:85–100. 10.1016/j.addr.2017.07.009 28709936

[16] Abramovitz L, Rubinek T, Ligumsky H, Bose S, Barshack I, Avivi C, Kaufman B, Wolf I. KL1 internal repeat mediates klotho tumor suppressor activities and inhibits bFGF and IGF-I signaling in pancreatic cancer. Clin Cancer Res. 2011; 17:4254–66. 10.1158/1078-0432.CCR-10-2749 21571866

[17] Kwon SM, Kim SA, Yoon JH, Yook JI, Ahn SG. Global analysis of gene expression profiles in the submandibular salivary gland of klotho knockout mice. J Cell Physiol. 2018; 233:3282–94. 10.1002/jcp.26172 28885690 PMC5765504

[18] Kurosu H, Ogawa Y, Miyoshi M, Yamamoto M, Nandi A, Rosenblatt KP, Baum MG, Schiavi S, Hu MC, Moe OW, Kuro-o M. Regulation of fibroblast growth factor-23 signaling by klotho. J Biol Chem. 2006; 281:6120–23. 10.1074/jbc.C500457200 16436388 PMC2637204

[19] Olejnik A, Franczak A, Krzywonos-Zawadzka A, Kałużna-Oleksy M, Bil-Lula I. The Biological Role of Klotho Protein in the Development of Cardiovascular Diseases. Biomed Res Int. 2018; 2018:5171945. 10.1155/2018/5171945 30671457 PMC6323445

[20] Yamamoto M, Clark JD, Pastor JV, Gurnani P, Nandi A, Kurosu H, Miyoshi M, Ogawa Y, Castrillon DH, Rosenblatt KP, Kuro-o M. Regulation of oxidative stress by the anti-aging hormone klotho. J Biol Chem. 2005; 280:38029–34. 10.1074/jbc.M509039200 16186101 PMC2515369

[21] Doi S, Zou Y, Togao O, Pastor JV, John GB, Wang L, Shiizaki K, Gotschall R, Schiavi S, Yorioka N, Takahashi M, Boothman DA, Kuro-o M. Klotho inhibits transforming growth factor-beta1 (TGF-beta1) signaling and suppresses renal fibrosis and cancer metastasis in mice. J Biol Chem. 2011; 286:8655–65. 10.1074/jbc.M110.174037 21209102 PMC3048747

[22] Hu MC, Shi M, Zhang J, Pastor J, Nakatani T, Lanske B, Razzaque MS, Rosenblatt KP, Baum MG, Kuro-o M, Moe OW. Klotho: a novel phosphaturic substance acting as an autocrine enzyme in the renal proximal tubule. FASEB J. 2010; 24:3438–50. 10.1096/fj.10-154765 20466874 PMC2923354

[23] Hu MC, Shi M, Zhang J, Quiñones H, Griffith C, Kuro-o M, Moe OW. Klotho deficiency causes vascular calcification in chronic kidney disease. J Am Soc Nephrol. 2011; 22:124–36. 10.1681/ASN.2009121311 21115613 PMC3014041

[24] Metzler MA, Venkatesh SG, Lakshmanan J, Carenbauer AL, Perez SM, Andres SA, Appana S, Brock GN, Wittliff JL, Darling DS. A systems biology approach identifies a regulatory network in parotid acinar cell terminal differentiation. PLoS One. 2015; 10:e0125153. 10.1371/journal.pone.0125153 25928148 PMC4416001

[25] Yoon HS, Chen X, Yang VW. Kruppel-like factor 4 mediates p53-dependent G1/S cell cycle arrest in response to DNA damage. J Biol Chem. 2003; 278:2101–05. 10.1074/jbc.M211027200 12427745 PMC2229830

[26] Zhang W, Geiman DE, Shields JM, Dang DT, Mahatan CS, Kaestner KH, Biggs JR, Kraft AS, Yang VW. The gut-enriched Kruppel-like factor (Kruppel-like factor 4) mediates the transactivating effect of p53 on the p21WAF1/Cip1 promoter. J Biol Chem. 2000; 275:18391–98. 10.1074/jbc.C000062200 10749849 PMC2231805

[27] Shie JL, Chen ZY, Fu M, Pestell RG, Tseng CC. Gut-enriched Krüppel-like factor represses cyclin D1 promoter activity through Sp1 motif. Nucleic Acids Res. 2000; 28:2969–76. 10.1093/nar/28.15.2969 10908361 PMC102679

[28] Yoon HS, Yang VW. Requirement of Krüppel-like factor 4 in preventing entry into mitosis following DNA damage. J Biol Chem. 2004; 279:5035–41. 10.1074/jbc.M307631200 14627709 PMC1262649

[29] Fonseca I, Moura Nunes JF, Soares J. Expression of CD44 isoforms in normal salivary gland tissue: an immunohistochemical and ultrastructural study. Histochem Cell Biol. 2000; 114:483–88. 11201610 10.1007/s004180000220

[30] Maria OM, Maria AM, Cai Y, Tran SD. Cell surface markers CD44 and CD166 localized specific populations of salivary acinar cells. Oral Dis. 2012; 18:162–68. 10.1111/j.1601-0825.2011.01858.x 21973167

[31] Sato A, Okumura K, Matsumoto S, Hattori K, Hattori S, Shinohara M, Endo F. Isolation, tissue localization, and cellular characterization of progenitors derived from adult human salivary glands. Cloning Stem Cells. 2007; 9:191–205. 10.1089/clo.2006.0054 17579552

[32] Kim SA, Lam TG, Yook JI, Ahn SG. Antioxidant modifications induced by the new metformin derivative HL156A regulate metabolic reprogramming in SAMP1/kl (-/-) mice. Aging (Albany NY). 2018; 10:2338–55. 10.18632/aging.101549 30222592 PMC6188477

[33] Lukasova V, Buzgo M, Sovkova V, Dankova J, Rampichova M, Amler E. Osteogenic differentiation of 3D cultured mesenchymal stem cells induced by bioactive peptides. Cell Prolif. 2017; 50:e12357. 10.1111/cpr.12357 28714176 PMC6529093

