# Supplementary material for: Soluble klotho regulates the function of salivary glands by activating KLF4 pathways
Source: Aging (Albany NY). 2019 Oct 2;11(19):8254–69. doi: 10.18632/aging.102318 (PMC6814581; doi:10.18632/aging.102318)
Supplement: Supplementary Figures [file aging-11-102318-s001.pdf]

SUPPLEMENTARY FIGURES

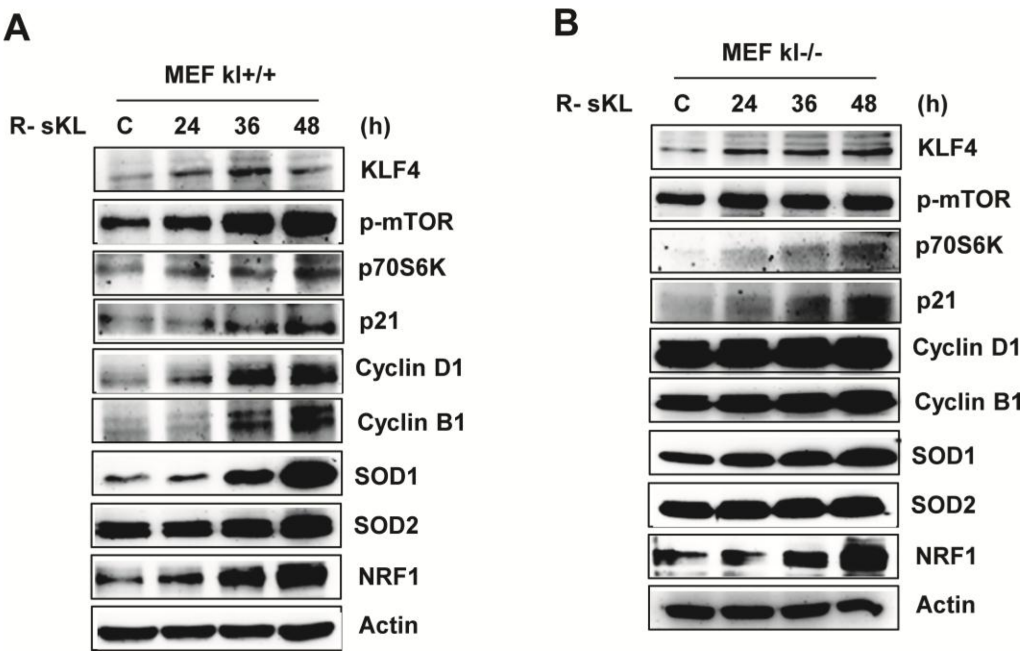

Supplementary Figure 1. Effects of soluble klotho protein on the expression of proteins belonging to the KLF4 pathway in wild-type and klotho (-/-) MEFs. (**A**, **B**) The expression of proteins related to the KLF4 pathway. Wild-type and klotho (-/-) MEFs were treated with soluble klotho protein (0.3 μg) at the indicated times. Western blot analysis was performed to assess the KLF4, mTOR, p70S6K, p21, NRF2, cyclin D1, cyclin B1, SOD1, and SOD2 levels.

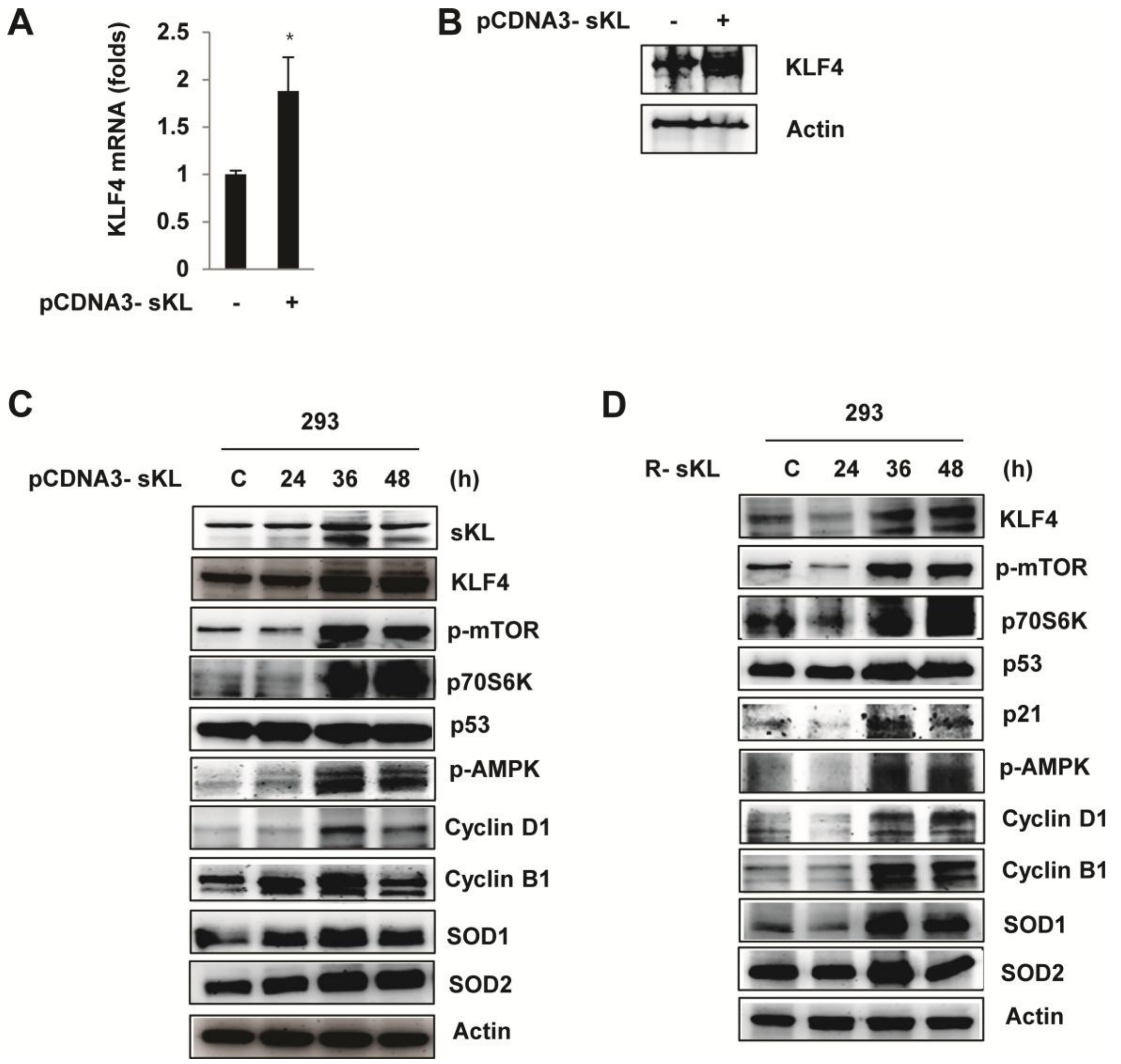

Supplementary Figure 2. The expression of KLF4 in HEK293 cells. (**A**) qRT-PCR analysis of KLF4 in soluble klotho-expressing HEK293 cells. (**B**) Total protein samples were prepared in soluble klotho-transfected HEK293 cells, followed by Western blot analysis to examine the expression levels of KLF4. (**C**, **D**) Western blot analysis was performed to assess the KLF4- and KLF4-related protein levels in soluble klotho-transfected or soluble klotho protein-treated cells.

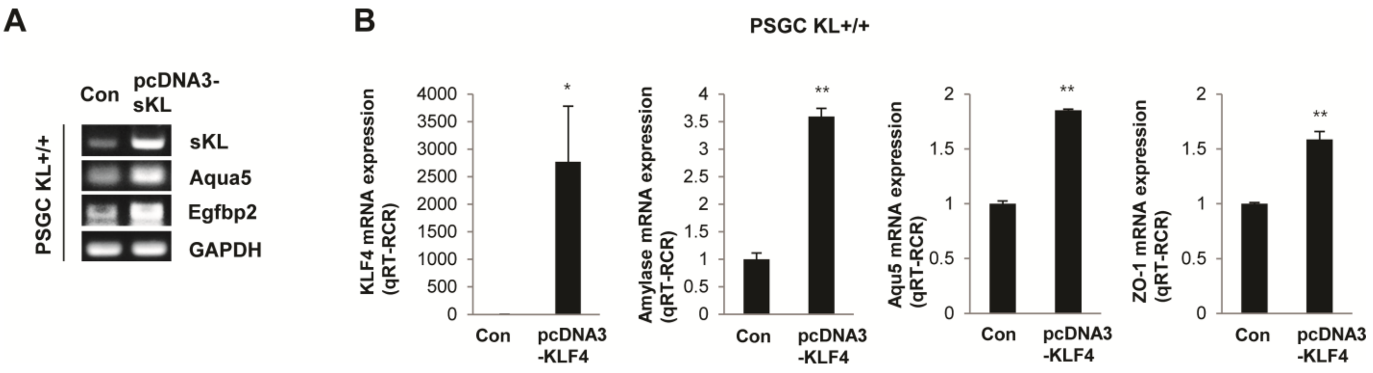

Supplementary Figure 3. The expression of salivary gland functional proteins induced by KLF4 in primary salivary gland cells (PSGCs). The mRNA expression of functional proteins (aquaporin 5, EGFBP2, ZO-1 and amylase) in KLF4-overexpressing KL(+/+) PSGCs as determined by RT-PCR (**A**) and qRT-PCR analyses (**B**).

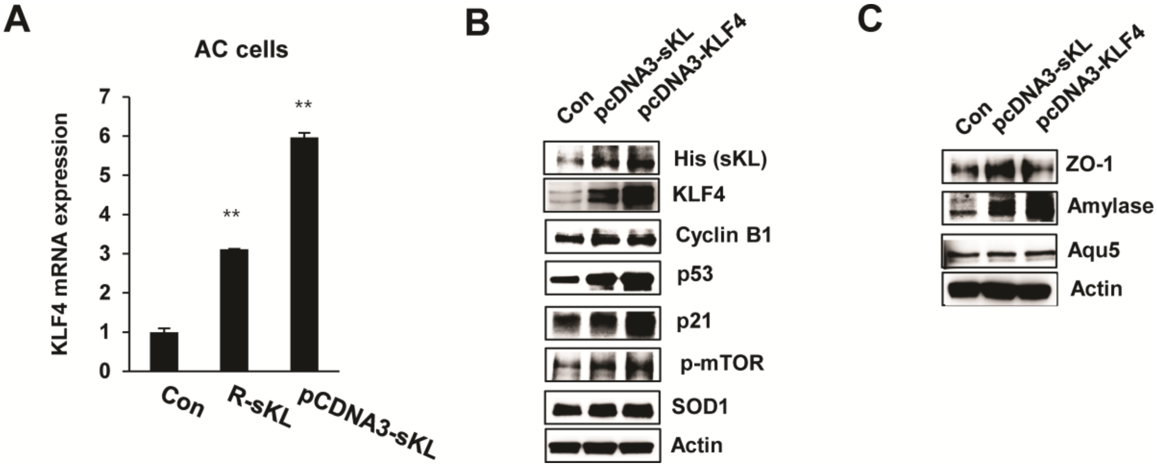

Supplementary Figure 4. The expression of salivary gland functional proteins in acinar cells (ACs). (**A**) qRT-PCR analysis of KLF4 mRNA in soluble klotho- or KLF4-transfected ACs. (**B**) The expression of proteins related to the KLF4 pathway. (**C**) The mRNA expression of functional proteins (aquaporin 5, EGFBP2, ZO-1 and amylase) in soluble klotho- or KLF4-overexpressing ACs as determined by Western blot.
